# Mediation analysis with a time-to-event outcome: a review of use and reporting in healthcare research

**DOI:** 10.1186/s12874-018-0578-7

**Published:** 2018-10-29

**Authors:** Lauren Lapointe-Shaw, Zachary Bouck, Nicholas A. Howell, Theis Lange, Ani Orchanian-Cheff, Peter C. Austin, Noah M. Ivers, Donald A. Redelmeier, Chaim M. Bell

**Affiliations:** 1 Department of Medicine, University of Toronto, Toronto, Canada; 2 Institute for Health Policy, Management and Evaluation, University of Toronto, Toronto, Canada; 3 Institute for Clinical Evaluative Sciences, Toronto, Canada; 4 Institute for Health Systems Solutions and Virtual Care, Women’s College Hospital, Toronto, Canada; 5 Centre for Urban Health Solutions, St. Michael’s Hospital, Toronto, Canada; 6 Section of Biostatistics, University of Copenhagen, Copenhagen, Denmark; 7 Center for Statistical Science, Peking University, Beijing, China; 8 Library and Information Services, University Health Network, Toronto, Canada; 9 Department of Family and Community Medicine, University of Toronto, Toronto, Canada; 10 Department of Medicine, Mount Sinai Health System, Toronto, Canada

**Keywords:** Mediation, Indirect effect, Counterfactuals, Reporting, Mediation analysis, Survival, Time-to-event, Methodology

## Abstract

**Background:**

Mediation analysis tests whether the relationship between two variables is explained by a third intermediate variable. We sought to describe the usage and reporting of mediation analysis with time-to-event outcomes in published healthcare research.

**Methods:**

A systematic search of Medline, Embase, and Web of Science was executed in December 2016 to identify applications of mediation analysis to healthcare research involving a clinically relevant time-to-event outcome. We summarized usage over time and reporting of important methodological characteristics.

**Results:**

We included 149 primary studies, published from 1997 to 2016. Most studies were published after 2011 (*n* = 110, 74%), and the annual number of studies nearly doubled in the last year (from *n* = 21 to *n* = 40). A traditional approach (causal steps or change in coefficient) was most commonly taken (*n* = 87, 58%), and the majority of studies (*n* = 114, 77%) used a Cox Proportional Hazards regression for the outcome. Few studies (*n* = 52, 35%) mentioned any of the assumptions or limitations fundamental to a causal interpretation of mediation analysis.

**Conclusion:**

There is increasing use of mediation analysis with time-to-event outcomes. Current usage is limited by reliance on traditional methods and the Cox Proportional Hazards model, as well as low rates of reporting of underlying assumptions. There is a need for formal criteria to aid authors, reviewers, and readers reporting or appraising such studies.

**Electronic supplementary material:**

The online version of this article (10.1186/s12874-018-0578-7) contains supplementary material, which is available to authorized users.

## Background

**Fig. 1:**
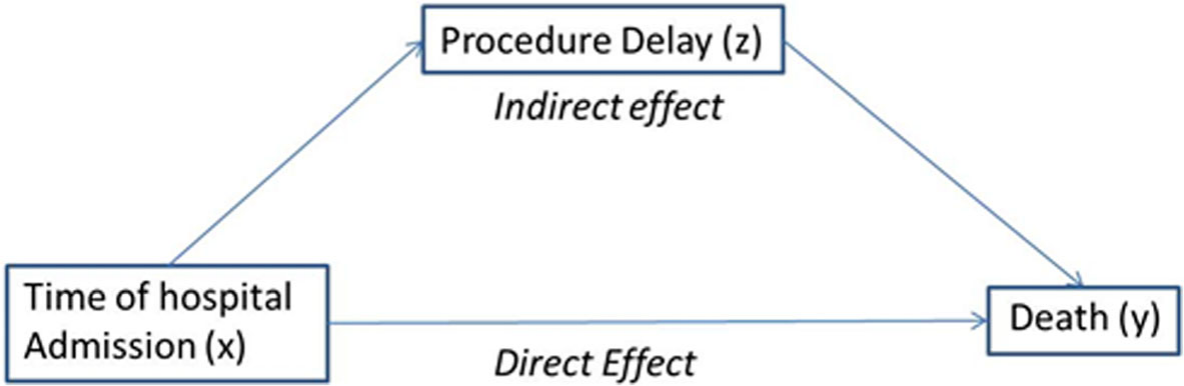
Causal diagram depicting the relationship between independent (x), dependent (y), and mediator (z) variables

**Fig. 2:**
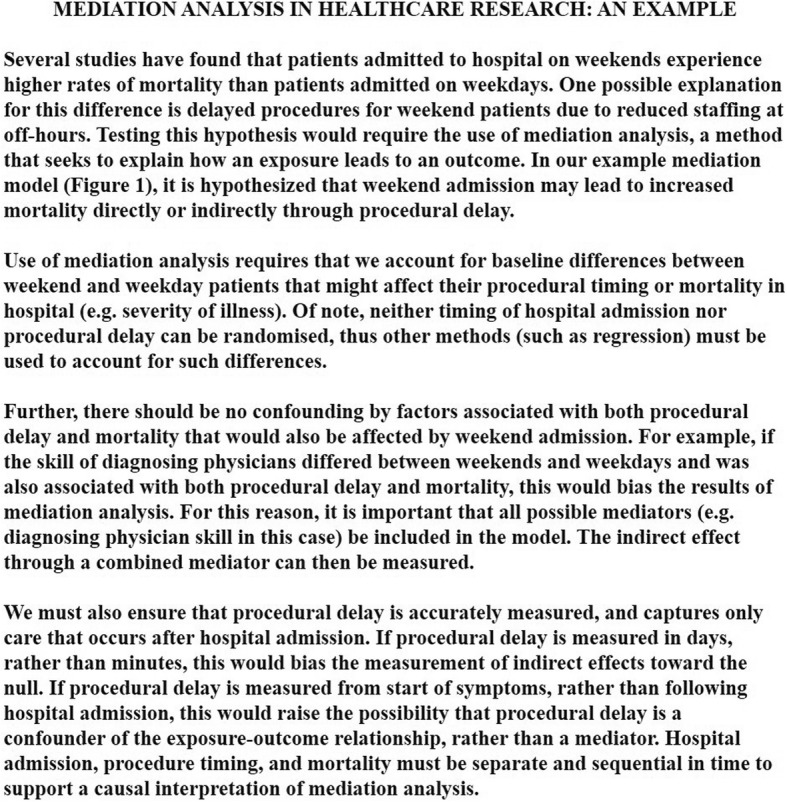
An example of mediation analysis in healthcare research

Mediator variables lie along the causal pathway between an independent and dependent variable, explaining all or part of the effect of the independent variable on the dependent variable [1]. While mediation analysis has been prominently featured in social science research, this methodology is now gaining popularity in healthcare research. It is used primarily for two purposes: to understand how certain relationships (including treatment effects) occur, and to identify possible targets for future interventions [1]. A test of mediation examines whether the effect of the independent variable (x) on the dependent variable (y) occurs via a third, intervening variable (z) (see Figs. 1, 2). This basic structure – referred to as a single-mediator model – can be expanded to include additional considerations such as multiple mediators and moderated mediation [2–5].

The causal interpretation implicit in any mediation analysis rests on a number of untestable assumptions, which are often underreported in published research [6, 7]. In particular, the *sequential ignorability* assumption states that there is no unmeasured confounding of the exposure-mediator, mediator-outcome, or exposure-outcome relationships [8]. Furthermore, there must be no confounders (measured or unmeasured) of the mediator-outcome that depend on the exposure [9–11]. While these assumptions can theoretically be satisfied by random allocation, it is not possible to randomise both exposure and mediator [12]. As a consequence, some suggest that any mediation analysis be accompanied by sensitivity analyses to investigate the robustness of findings to violations of this crucial assumption [8]. Furthermore, tests of mediation assume that the mediator has been appropriately defined and measured [13]. In addition to these fundamental assumptions, the temporal sequence of independent variable, mediator, and dependent variable should support the argument for causation [14, 15].

Traditional methods of mediation analysis include fulfilling a series of stepwise criteria (causal steps), as proposed by Baron and Kenny in 1986 [16]. To quantify the degree of mediation, simple formulas combine parameter estimates obtained from a series of regressions [1, 17, 18]. The resulting difference and product tests were originally intended for linear relationships with continuous outcomes such as blood pressure, but have been adapted for binary outcomes such as mortality. Unfortunately these methods are ill-adapted to non-normally distributed continuous and/or censored variables, such as time-to-event outcomes [14].

Mortality and survival time are a major focus in healthcare research. Survival analysis allows investigators to study these important outcomes with appropriate consideration for variable follow-up times, censoring, and competing risks. Cox Proportional Hazards (PH) regression is commonly used for such analyses, yet its use in mediation analysis poses some important challenges. The semi-parametric Cox model builds on proportionality of the hazards. Proportionality is violated when adding an additional (mediator) variable to a correctly specified Cox regression model. This addition could shift the baseline hazards up or down, rather than only altering the slope of the hazard function [19]. Statisticians term this phenomenon the “non-collapsibility” of the hazard ratio [20]. As a result, parameter estimates obtained with and without a mediator cannot be meaningfully compared as they might be in a linear model [21, 22]. This problem is exacerbated as the outcome frequency rises. Thus, use of Cox PH regression to approximately estimate indirect effects via difference or product of coefficients rests on the assumption that the outcome is rare [21]. Parametric survival (including accelerated failure time) and additive hazard models do not have this limitation [14, 21]. These models provide readily interpretable outcome measures (expressed as hazard ratios or differences), yet they are less familiar to clinical researchers than the popular Cox model [14].

Path analysis provides another possible approach, and allows for modelling of the relationships between a large number of confounding and mediator variables [23]. Structural Equation Modelling (SEM) is derived from path analysis, and incorporates latent variables, allowing uncertainty of variable measurement to be incorporated into the analysis [23]. Using SEM and path analysis, relationships can be deconstructed into subcomponents and indirect effects obtained [24]. Although these models depend on linearity of relationships, time-to-event outcomes can be modelled in SEM and path using discrete time survival analysis or dynamic path analysis, wherein the follow-up period is broken down into short time intervals [25–28]. Such methods allow mediation effects to be expressed as hazard ratios and hazard differences, respectively. Drawing conclusions based on results of SEM and path analysis depends on adequate linear model specification, and that all included variables are free of unmeasured confounding [29].

As a result of the linearity assumptions inherent to previous methods of mediation analysis, alternative methods have been sought. The counterfactual or potential outcomes approach evolved more recently from the literature on causal inference [30]. In this framework, mediation analysis is treated as a problem of missing data, and observed and unobserved potential outcomes are modelled. This flexible approach can accommodate any data distribution, and be applied to any type of mediator or outcome variable, including time-to-event [8]. In addition to meeting the assumptions underlying a causal interpretation of mediation analysis, implementation of this approach requires meeting the assumptions inherent to any selected models. Within the counterfactual framework, additive hazard, parametric survival and marginal structural models also allow for measurement of indirect effects, without the limitation to rare outcomes [31].

While the above approaches offer a range of strategies to address mediation analysis with a time-to-event outcome, some require advanced statistical coding, or at least an understanding of counterfactual concepts. While mediation analysis is increasingly utilized, we do not know how healthcare researchers have addressed this problem. Although others have described the recent reporting of causal mediation analysis, they have not examined practices specific to time-to-event outcomes, nor have they described temporal trends in the use of these methods [6, 7]. We sought to evaluate the usage and reporting of mediation analysis with time-to-event outcomes in all published healthcare research.

## Methods

### Systematic search and screening

A systematic and sensitive search strategy, developed with a research librarian (AOC), was used to identify published articles employing mediation analysis with a time-to-event outcome. The search strategy was initially developed for Ovid Medline, and then customized for use in the other databases. At the time of the search, specific subject headings for mediation analysis and time-to-event were unavailable in the databases used. As a result, the strategy was devised using an extensive list of appropriate text words and phrases mined from sample articles and through input from subject specialists on the team. Ovid Medline, Ovid Medline Epub Ahead of Print and In-Process & Other Non-Indexed Citations, and Ovid Embase were searched from inception to date of search. All searches were executed between December 9th and 12th, 2016. No limits for date were applied and animal-only studies were excluded where applicable. Book and conference materials were also excluded from Embase. In addition, cited reference searches were conducted in the Web of Science Core Collection for any articles citing one of five highly cited and relevant methodological articles [8, 31–34] (see Additional file 1 for details of search).

Studies relating to human healthcare, with an empiric application of mediation analysis and a clinically relevant time-to-event outcome, were selected for inclusion. Since we were most interested in how a non-specialist healthcare researcher applied the methodology, theoretical papers with an illustrative application were excluded. Review articles were manually searched for relevant primary studies.

Inclusion criteria were pre-specified and refined after pilot screening of 10 full-text articles. Specifically, inclusion criteria were refined to include a formal test of mediation, in the form of meeting specific listed criteria (e.g. directly cite Baron and Kenny or describe causal steps methodology), a statistical test with a *p*-value, or a measurement of indirect effect/proportion mediated. This was necessary as many studies did not set out to assess mediation, but mentioned it as a possible explanation for weakening of an observed association upon the introduction of other variables.

All eligible abstracts were screened in duplicate by LLS and ZB. Abstracts deemed eligible by either LLS or ZB were included for full-text review. All full texts were screened by LLS. Uncertainty in study inclusion or extraction was addressed by discussion with TL, a methodological expert. NAH performed duplicate full-text screening and extraction of a 10% random sample (*n* = 33) in order to assess reproducibility.

Duplicate screening showed 82% agreement, Cohen’s kappa was 0.63 (95% CI 0.36–0.89). Disagreements related to the relevance of a clinical outcome (sick leave, *n* = 1, study was included) and whether a formal test of mediation was described (*n* = 5, all excluded). Though these five excluded studies did not explicitly state how they assessed mediation or indirect effects, they appeared to use the following strategies: partial causal steps (*n* = 1), change in coefficient (*n* = 4). They all used Cox PH models for the outcome, and none mentioned any of the assumptions fundamental to mediation analysis.

### Extraction

The criteria for extraction were developed in consideration of the STROBE statement [35], existing systematic reviews of mediation analysis [6, 7, 36], and methodological concerns unique to time-to-event outcomes.

After a pilot extraction from 10 full-text articles, extraction criteria were refined and all extraction performed by LLS. Where studies included a methodological supplement for mediation details, these were also reviewed for relevant information. The results of duplicate extraction from a 10% random sample of included studies are presented in Table 1. The criteria tested for inter-rater reliability were pre-specified based on their importance. Estimates of Cohen’s kappa (with 95% confidence intervals) were obtained using the “kappa2” function in the “irr” package in R [37].

**Table 1:** Agreement on important characteristics, at re-extraction of a 10% random sample of included studies

Characteristic	Unweighted Cohen’s Kappa (95% CI)
Funding source	0.75 (0.5–1)
Study Design	0.65 (0.02–1)
Type of analysis (confirmatory/hypothesis-based versus exploratory)	0.14 (0–0.5)
Mediation analysis is primary aim of study	0.52 (0.12–0.92)
Causal diagram included	1 (1–1)
Sample size	0.75 (0.55–0.96)
Power/sample size calculation included	0 (unable to estimate, too infrequent)
Method of mediation analysis	0.84 (0.83–1)
Type of time-to-event model	0.91 (0.75–1)
Competing risks considered	0 (unable to estimate, too infrequent)
If clustering of data, was this addressed in the analysis?	0.61 (0.15–1.0)
Outcome frequency > =10%	0.79 (0.54–1)
Rare outcome limitation for Cox model mentioned	Unable to estimate, all false (100% agreement)
Temporal separation clearly defined	0.76 (0.47–1)
No unmeasured confounding of exposure/outcome	0.82 (0.49–1)
No unmeasured confounding of mediator/outcome	0.85 (0.57–1)
No unmeasured confounding of exposure/mediator	0.6 (0.13–1)
No exposure-dependent confounding of mediator-outcome	0.64 (0–1)
Accurate measurement of mediator	0.65 (0.32–0.99)
Interaction between exposure and mediator considered/tested	0.6 (0.19–1)
Was a method used to address confounding of exposure or mediator?	N/A (100% used regression for both exposure and mediator, 100% agreement)
Sensitivity analysis relating to mediation analysis	0.44 (0.05–0.75)
Measures reported-indirect effect	0.76 (0.46–1)
Measures reported- proportion mediated	0.86 (0.61–1)
Precision estimate for indirect effect	1.0 (N/A)
Precision estimate for proportion mediated	0.77 (0.34–1)

We extracted information on study characteristics including methodological approach to mediation analysis, statistical analysis, assumptions addressed, and measures reported. Results are presented as counts and frequencies for categorical or binary characteristics, and as median and interquartile range for study sample size.

As suggested by a peer reviewer, we added selected comparisons of studies published before or after 2013. Comparisons were made with the Chi-square test, with *p* < 0.05 defined as significant; Fischer’s exact text was used for comparisons where frequencies of 0 (empty cells) were reported.

## Results

**Fig. 3:**
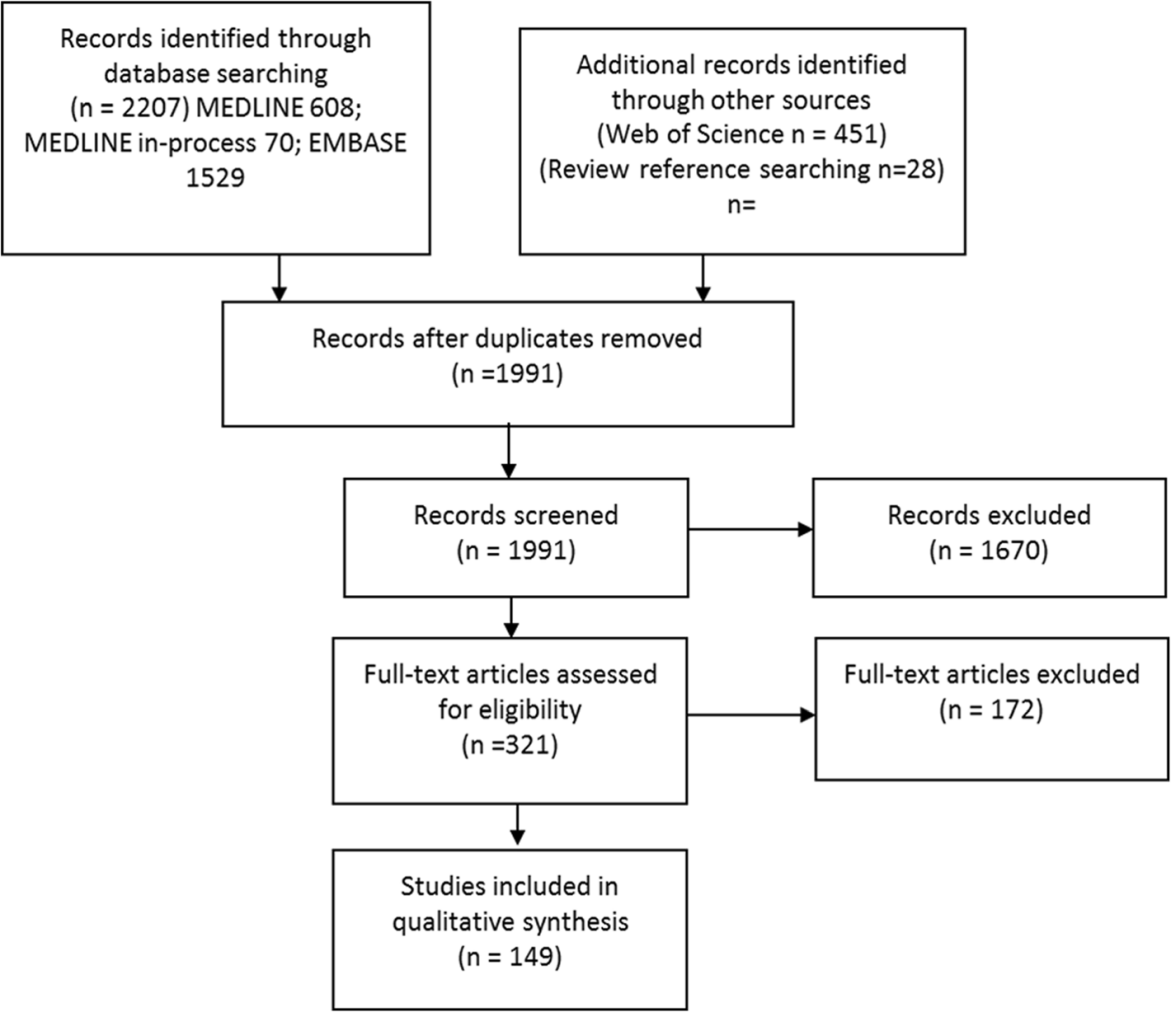
PRISMA Flow Diagram

Our search yielded 1991 unique abstracts, of which 321 were selected for further review (see Fig. 3). Of these, 8 were excluded as they did not relate to human healthcare, 110 because they did not include mediation criteria, test, or measurement of the indirect effect/proportion mediated. Another 12 were excluded because they did not include a clinically meaningful outcome, and 41 because the outcome of mediation analysis was not time-to-event. Further, one full text could not be reviewed as it was in Arabic. This left 149 studies eligible for extraction (see Additional file 2 for the list of included studies).

**Fig. 4:**
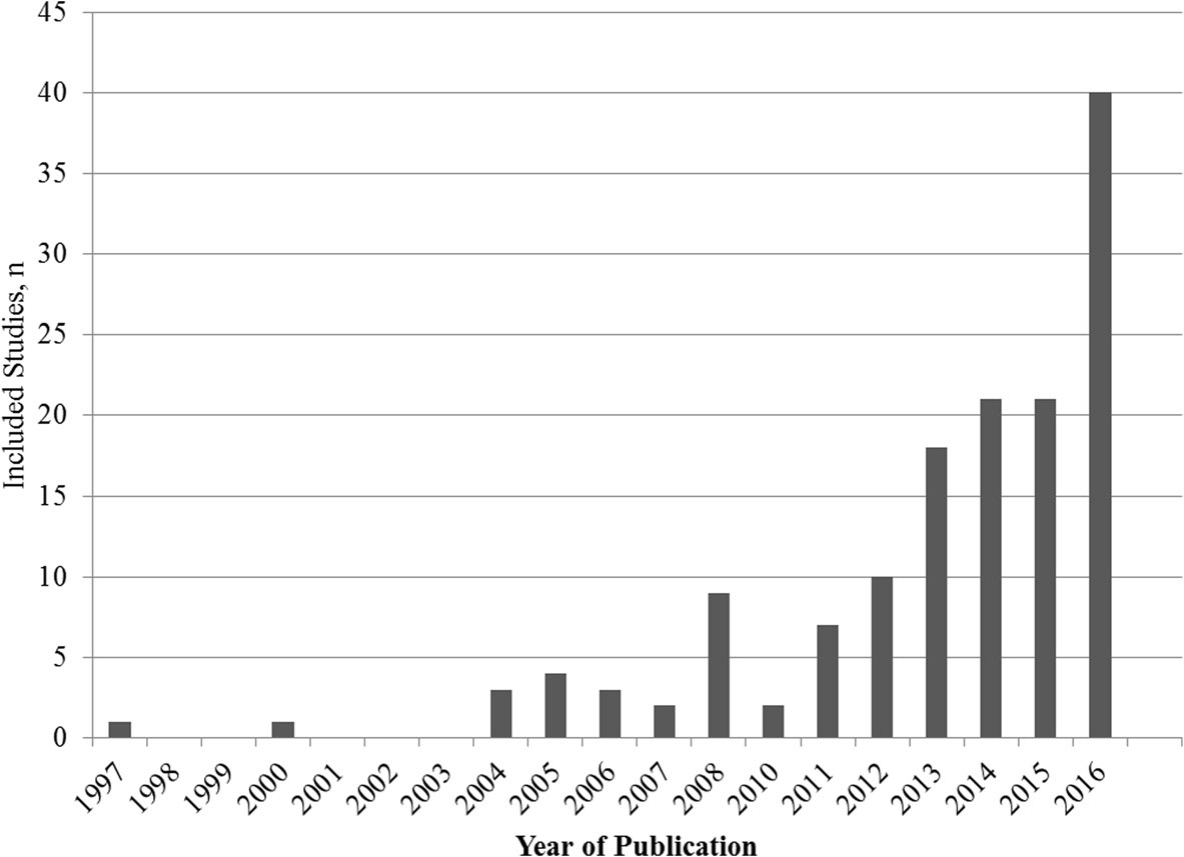
Included studies, by year of publication

The earliest study was published in 1997, and there were fewer than 10 studies per year up to 2011. There were 110 included studies (74%) published in 2012 or later, and the number of studies nearly doubled from 2015 to 2016 (*n* = 21 in 2015, *n* = 40 in 2016, see Fig. 4). Over half of included studies had a first author based in the United States (*n* = 77, 51%), and 82 were from North America (55%). Otherwise, 55 (37%) publications originated in Europe, 5 (3%) were from Asia, 5 (3%) from Australia, 1 (< 1%) was from Israel and 1 (< 1%) from Brazil. Sixty-four individuals were listed as an author on more than one included study. The number of studies per author ranged from 1 to 6, with 8 individuals listed on 5 or more studies. Included studies most commonly came from journals in the areas of epidemiology (*n* = 37, 25%), psychology/psychiatry (*n* = 19, 13%), cardiology (*n* = 17, 11%), oncology (*n* = 13, 9%), and general medicine (n = 13, 9%). Eleven studies (7%) were published in high impact journals (impact factor 10 or greater) [38].

Mediation analysis was in most cases (*n* = 80, 54%) not the primary study aim, and was frequently an exploratory analysis (*n* = 74, 50%, see Table 2). Many studies (*n* = 76, 51%) tested multiple mediators. The most commonly tested mediators were psychological or psychiatric (*n* = 32, 21%), physiologic parameters (*n* = 34, 23%) or lifestyle factors (*n* = 31, 21%). The majority of mediators were continuous (*n* = 60, 40%) or binary (*n* = 56, 38%) variables. The most common outcome was the onset of a new medical condition or exacerbation of an existing condition (*n* = 68, 46%). A causal diagram was included in a third (*n* = 59, 40%) of studies. Results supporting a mediation model were reported in 130 studies (87%), and 19 studies (13%) reported that all tested mediators either did not meet criteria or were statistically not significant. Sixty-four studies (43%) reported mixed results (both significant and not significant) for the various mediators being tested.

**Table 2:** Characteristics of mediation analyses with time-to-event outcome in healthcare research, *n* = 149

Included study characteristic	Result
Funding source, *n* (%)	
Government	113 (76)
Foundation	37 (25)
Hospital	6 (4)
Industry	6 (4)
University	4 (3)
Professional association	1 (< 1)
None stated	13 (9)
Study design, *n* (%)	
Cohort	131 (88)
Randomised Controlled Trial	8 (5)
Case-cohort	5 (3)
Case control	4 (3)
Cross-sectional	1 (< 1)
Type of analysis, *n* (%)	
Confirmatory/Hypothesis-based	72 (48)
Exploratory	74 (50)
Not able to infer	3 (2)
Mediation analysis is primary aim of study, *n* (%)	69 (46)
Multiple mediators tested, *n* (%)	76 (51)
Type of mediator, *n* (%)	
Continuous	60 (40)
Binary	56 (38)
Categorical	25 (17)
Interval/Ordinal	25 (17)
Latent	8 (5)
Most common content of mediator^a^, *n* (%)	
Physiologic (e.g. blood pressure, heart rate, weight)	34 (23)
Psychological/psychiatric	32 (21)
Lifestyle (e.g. alcohol, smoking, nutrition, exercise, sleep)	31 (21)
Biomarker (blood test results)	24 (16)
Health	17 (11)
Comorbidity	13 (9)
Treatment	8 (5)
Functioning	8 (5)
Socioeconomic	8 (5)
Environment	6 (4)
Reproductive	2 (1)
Most common outcomes, *n* (%)	
New medical condition or exacerbation of an existing condition	68 (46)
All-cause mortality	48 (32)
Cause-specific mortality	21 (14)
Disability or sick leave	6 (4)
Causal diagram included, *n* (%)	
Causal steps/change in coefficient (*n* = 87)	22 (25)
Counterfactuals (*n* = 32)	16 (50)
SEM/path (*n* = 23)	18 (78)
Product of coefficients (*n* = 6)	3 (50)
Cannot infer (*n* = 1)	0 (0)
Sample size, median (IQR)	3345 (637–16,061)
Power/sample size, *n* (%)	
Calculation	1 (< 1)
Consideration	3 (2)
Method of mediation analysis, *n* (%)	
Causal steps, including Baron-Kenny	41 (28)
Change in coefficient in a single regression	46 (31)
Counterfactuals	32 (21)
SEM/path	23 (15)
Product of coefficients	6 (4)
Cannot infer	1 (< 1)
Statistical tests for no mediation/indirect effect, *n* (%)	
Sobel	7 (5)
Other product test	14 (9)
Difference test	2 (1)
Z-test of mediated proportion	1 (< 1)
Joint significance test	1 (< 1)
Olaf & Finn test	1 (< 1)
Type of time-to-event model, *n* (%)	
Cox proportional hazard	114 (77)
Additive hazard	10 (7)
Linear	7 (5)
Discrete time survival model	6 (4)
Failure time/parametric survival	5 (3)
Marginal structural model	3 (2)
Log linear Poisson	1 (< 1)
Quantile regression	1 (< 1)
Cannot infer	5 (3)
Specific mediation software mentioned, *n*	
Causal steps/change in coefficient	
SAS “mediate” macro	2
PRODCLIN	1
Counterfactuals	
R	8
R “mediation”	2
SAS	1
SAS “mediate” macro	1
STATA “medeff”	1
SEM/path	
Mplus	13
SAS	1
STATA mediation package	1
LISREL	1
Competing risks considered, *n* (%)	4 (3)
If clustering of data, was this addressed in the analysis? *n* (%)	
Not multilevel	114
Yes	19 (54)
No	8 (23)
Cannot determine	8 (23)
Cox models, outcome frequency^b^, *n* (%)	
> or equal to 5%	74 (65)
> or equal to 10%	55 (48)
Rare outcome limitation for Cox model mentioned^b^, *n* (%)	8 (7)
Temporal separation clearly defined, *n* (%)	
Yes	37 (25)
Overlap exposure and mediator	89 (60)
Overlap mediator/outcome	7 (5)
Cannot determine	19 (13)
Acknowledged as a limitation	20 (13)
Mediation assumptions (or limitation) stated, *n* (%)	
No unmeasured confounding of exposure/outcome	29 (19)
No unmeasured confounding of mediator/outcome	29 (19)
No unmeasured confounding of exposure/mediator	22 (15)
No exposure-dependent confounding of mediator-outcome	17 (11)
Accurate measurement of mediator	31 (21)
Interaction between exposure and mediator considered/tested, *n* (%)	46 (31)
Method to address confounding of exposure (more than one can be used), *n* (%)	
Regression/modelling	137 (92)
Stratification/restriction	14 (9)
Randomisation	6 (4)
None	9 (6)
Method to address confounding of mediator (more than one can be used), *n* (%)	
Regression/modelling	138 (93)
Weighting	13 (9)
Stratification/restriction	13 (9)
Matching	1 (< 1)
None	10 (7)
Sensitivity analysis related to mediation analysis, *n* (%)	
Any	25 (17)
Confounding	8 (5)
Accurate measurement/specification of mediator	7 (5)
Temporal sequence assumption	6 (4)
Testing a combined mediator or all mediators in same model	5 (3)
Interaction/moderation	2 (1)
Measures of mediation reported, *n* (%)	
Causal steps/change in coefficient method (*n* = 87)	
Indirect effect	7 (8)
Proportion mediated	52 (60)
Counterfactuals (*n* = 32)	
Indirect effect	29 (91)
Proportion mediated	22 (69)
SEM/path (*n* = 23)	
Indirect effect	16 (70)
Proportion mediated	5 (22)
Other (*n* = 7)	
Indirect effect	3
Proportion mediated	4
Measures of precision reported, *n* (%)	
Causal steps/change in coefficient (*n* = 87)	
Indirect effect confidence interval	6 (7)
Proportion mediated confidence interval	17 (20)
Statistical test p-value or equivalent	10 (11)
Counterfactuals (*n* = 32)	
Indirect effect confidence interval	29 (91)
Proportion mediated confidence interval	14 (44)
SEM/path (*n* = 23)	
Indirect effect confidence interval	15 (65)
Proportion mediated confidence interval	2 (9)
Statistical test p-value or equivalent	4 (17)
Other (*n* = 7)	
Indirect effect confidence interval	3
Proportion mediated confidence interval	3
Statistical test p-value or equivalent	2

^a^Total exceeds 100% because of multiple mediators in many studies

^b^Denominator is 114

**Fig. 5:**
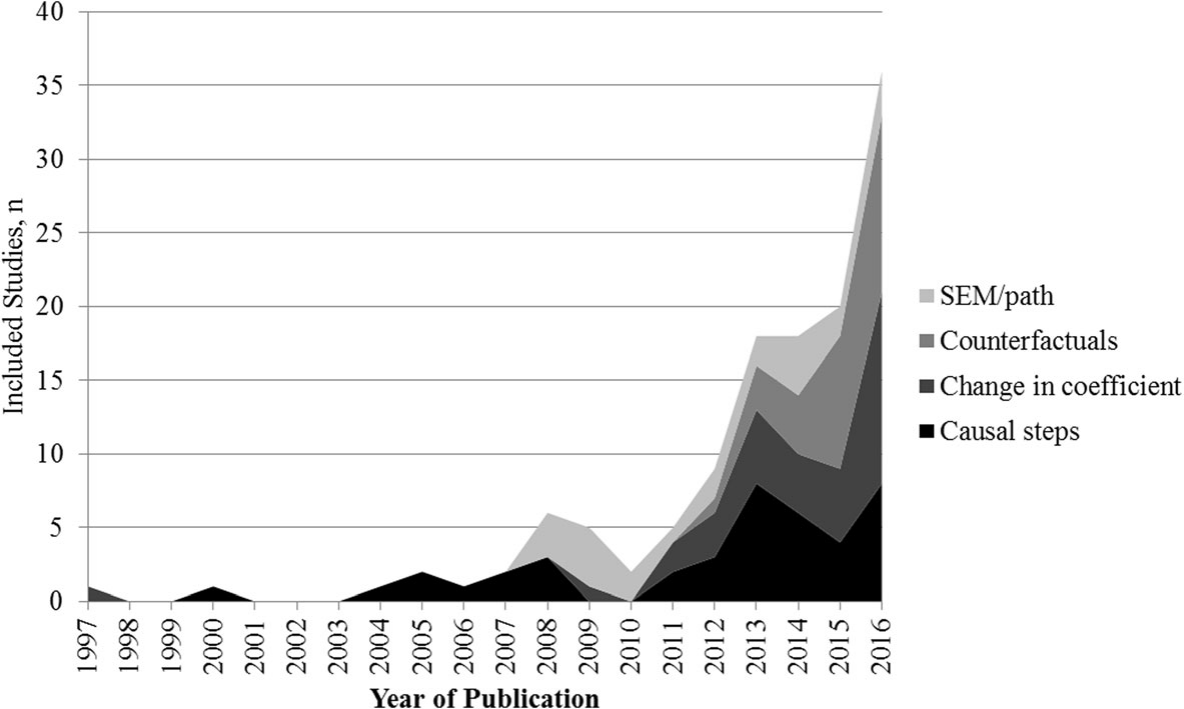
Included studies by year, according to their approach to mediation analysis

The most common method used for mediation analysis was comparing coefficients (henceforth known as “change in coefficient”) before and after a mediator was introduced into an exposure-outcome regression model without testing the other relationships included in the causal steps approach (*n* = 46, 31%). Other commonly used methods included causal steps (*n* = 41, 28%), counterfactuals (*n* = 32, 21%) and SEM or path analysis (*n* = 23, 15%). Studies published prior to 2010 predominantly featured causal steps and SEM/path approaches. After 2011, there was increased use of counterfactuals, change in coefficient, and causal steps methods of mediation analysis (see Fig. 5).

The majority (*n* = 136, 91%) of included studies described their funding source. In most cases (*n* = 113, 76%) this was governmental. The majority of included applications were cohort studies (*n* = 131, 88%). Most studies (*n* = 112, 75%) did not report exposure, mediator and outcomes that were clearly separated and sequential in time. Most commonly, overlap occurred in measurement of exposure and mediator (*n* = 89, 60%). The most common method used to deal with confounding of exposure and mediator was regression (*n* = 137, 92% for exposure; *n* = 138, 93% for mediator). Most studies did not mention any of the assumptions underlying mediation analysis (*n* = 97, 65%), a third (*n* = 52, 35%) mentioned at least one assumption, and eight (5%) mentioned all the assumptions. Among studies with a primary aim to assess mediation (*n* = 69), 33 (48%) mentioned one or more assumption, and six (9%) mentioned all assumptions. Sensitivity analysis relating to mediation analysis was included in 25 studies (17%).

Of 105 studies with outcomes other than all-cause mortality, four (4%) included consideration of competing risks. Of 35 studies with possible clustering of data in the exposure or mediator, 19 (54%) addressed this in their analysis. A third of studies (*n* = 46, 31%) mentioned or tested for interaction between exposure and mediator. Sample sizes ranged from 23 to 2,940,453, with 10 studies (7%) reporting sample sizes below 200. A single study included a sample size calculation, in this case for the association between the exposure and mediator [39], and another three studies discussed power and sample size as they relate to mediation. Software packages specifically used for mediation were mentioned in 32 studies (21%).

Indirect effect was reported in 55 studies (37%), proportion mediated in 83 studies (56%); 38 studies (26%) did not report either of these. Most studies reporting an indirect effect size included a measure of uncertainty (*n* = 53 of 55, 96%) around the estimate; however, only 36 studies (of 83, 43%) included a measure of uncertainty around the proportion mediated. A total of 16 studies (11%) included a test *p*-value, for the null hypothesis of no mediation.

The time-to-event outcome was most commonly modelled using a Cox PH model (*n* = 114, 77%). Only 7% (*n* = 8) of these included any mention of the rare outcomes assumption underlying use of this model. Of the 55 studies with a Cox PH model and an outcome frequency greater than 10%, 33 (60%) reported an estimate for either the indirect effect or the proportion mediated.

There were 49 studies published from 1997 to 2012, and 100 studies published from 2013 to 2016. More recently published studies were more likely to include measures of the indirect effect or proportion mediated (80% vs 63%, *p* = 0.03), a measure of precision such as a *p*-value or 95% CI (69% vs 45%, *p* = 0.005), and a sensitivity analysis relating to mediation (21% vs 8%, *p* = 0.049). In contrast, more recently published studies were not significantly more likely to contain mention of any (66% vs 63%, *p* = 0.7) or all assumptions (8% vs 0%, *p* = 0.053) underlying causal mediation analysis.

## Discussion

We studied the use and reporting of mediation analysis with a time-to-event outcome in healthcare research. We found that the use of mediation analysis with time-to-event outcomes increased over time and crossed multiple clinical fields. The most common time-to-event outcomes were the onset or exacerbation of a medical condition, and the most common mediators were physiologic, psychological or lifestyle factors. This suggests that researchers are most interested in understanding whether specific patient-related factors explain disease onset. Although included studies were a mix of exploratory and confirmatory/hypothesis-based, over half of included studies did not have mediation analysis as the primary aim. This indicates that mediation analysis is often used as an adjunct to help understand the findings of a primary research question. There were several instances of repeated authorship. This suggests further mechanistic exploration following an early discovery (for example, the research into premature death in the visually impaired, by Christ, Zheng, Lee and Lam [40–43]) as well as spread of the tools of mediation by a few highly collaborative methodological experts.

Included healthcare studies covered a broad range of mediation analysis practices. The majority of mediation analyses were undertaken using traditional methods (change in coefficient or causal steps). While the publication of seminal methodological articles in 2010–2012 can explain the growth in the number of studies using a counterfactual approach, the concurrent rise in use of traditional approaches suggests heightened awareness of broad mediation concepts among clinical researchers. Many researchers may prefer traditional approaches due to their intuitive appeal and easy implementation.

A minority of studies reported or discussed the assumptions underlying causal interpretations of mediation analysis, as described by others [7]. Many studies measured exposures and mediators simultaneously at baseline. When the mediator does not occur after the exposure, this weakens the argument for causation. Few studies mentioned assumptions relating to confounding, or accurate measurement of the mediator. When underlying assumptions go unmentioned, readers may mistakenly believe causal conclusions to be more robust than they actually are.

Most studies in our review used Cox PH regression to model a time-to-event outcome. In such cases, obtaining an estimate of the indirect effect depends on the outcome being rare. Where the outcome is common, measures of the indirect effect or proportion mediated will be incorrect [20]. Yet, Cox Proportional Hazards were often used to model a common outcome, and nearly two thirds of such studies reported one or both of these measures. Further, the rare outcome assumption was infrequently mentioned.

Our study identifies further details on current research practices. While regression methods were frequently used to adjust for baseline characteristics (potential confounders), few studies included any form of sensitivity analysis relating to mediation. Interaction of the exposure and mediator was most often not considered. A minority of studies addressed competing risks, which alter the interpretation of mediated effects where the outcome is other than all-cause mortality. Specifically, reported effects are only valid for the population that remains alive. Although sample sizes were generally large, only one study attempted to justify sample size, despite the existence of programs designed for this purpose [44, 45].

Although recent studies were more likely to include effect sizes, measures of precision (*p*-values or confidence intervals) and sensitivity analyses, reporting of characteristics and results of mediation analyses was overall suboptimal. The deficiencies identified in our study underscore the importance of developing standard reporting criteria for mediation analysis. Although others have made recommendations, no formal criteria have been published [7]. In addition to meeting established criteria for observational studies [35], we recommend that studies of mediation report the following items (see Table 3): whether mediation analysis is exploratory or confirmatory/hypothesis-based; the criteria used to assess mediation; the timing, measurement, and specification of exposure, mediator(s) and outcome variables; the type of model(s) and statistical software used; and methods used to account for any clustering or interactions between exposure and mediator. In addition, results reported should be accompanied by measures of precision (95% confidence intervals). Interpretation of the mediated effect should be made in the context of any competing risks (e.g. cause-specific indirect effect, among those who have not yet been censored). Assumptions underlying mediation analysis, and strategies used (regression, propensity scores, sensitivity analysis) to meet or test those assumptions should be detailed [9, 15]. Finally, the extent to which such assumptions limit causal inferences should be discussed in the limitations section.

**Table 3:** Reporting recommendations for mediation analysis with a time-to-event outcome

Section	Recommendation
Objectives	State whether mediation analysis(es) is/are exploratory or hypothesis-based
Methods	Specify criteria or statistical tests used to assess mediation, with references *Was the goal to categorize mediation as absent, partial or complete, or to estimate exact values for direct and indirect effects*?
	Detail how exposure, mediator and outcome variables were defined and measured
	Detail when exposure, mediator and outcome variables were measured
	Describe statistical models used for the mediator(s) and outcome(s), and any assumptions underlying use of such models (e.g. proportionality, rare outcome assumption for Cox Proportional Hazards models)
	State whether interaction between exposure and mediator was considered, and how
	Reference any software programs used for mediation analysis
	If relevant for exposure, mediator, and outcome being considered, state how the following were addressed: - clustering or repeated events - competing risks
	Describe assumptions underlying mediation analysis, and methods used to address these (e.g.: randomisation, regression, weighting, stratification, sensitivity analysis)
Results	Report measures of mediation effect (indirect effect or proportion mediated) accompanied by 95% confidence intervals
	Report p-values for mediation hypothesis testing
Discussion	Discuss limitations of causal inference based on mediation analysis results, including whether underlying assumptions were met Discuss magnitude and direction of any potential bias

In addition to these, mediation analyses should meet the STROBE criteria for observational studies [35]

We further recommend that researchers seeking to measure the degree of mediation or indirect effects avoid using a Cox PH model when the outcome is common (occurs in more than 10% of subjects). We suggest employing a counterfactual-based approach, which allows for mediators and outcomes of varied data distribution. Within this framework, the scale on which mediation is measured (hazard ratios, hazard differences) should be dictated by the medical problem at hand. Marginal structural, additive hazards and parametric survival models can be used when the outcome is common (> 10%). If familiarity and ease of implementation strongly favours a Cox-based approach, then authors must confirm that the outcome is rare.

Strengths of our study include its systematic search of multiple databases, and pre-defined extraction criteria. Further, previous systematic reviews of mediation analysis have been limited to specific journals or studies published in 2015 [6, 7]. While we were focused on mediation analysis with a time-to-event outcome, our inclusion of all methodological approaches over a long time frame has better illustrated the evolution of real-world research practices with this emerging methodology.

This study has several limitations. First, mediation analysis and time-to-event did not have specific index terms available in the databases searched, and thus we relied on keyword searching to identify eligible studies. We mitigated this by using a broad range of terms to maximize sensitivity. Second, our findings are limited to published studies. However, this was intentional as we were interested in understanding which practices would be accepted in the peer-reviewed literature. Third, it is possible that authors are not reporting their full approach to mediation analysis due to space limitations. This underscores the need for standard reporting criteria, in order to help authors, reviewers, and editors prioritize content.

## Conclusions

Mediation analysis for time-to-event outcomes is being used with increasing frequency by researchers around the world. There is ongoing reliance on traditional methods such as causal steps and change in coefficient. When combined with Cox PH modelling, these methods are limited to use with rare outcomes. As a result, methods using counterfactuals and/or alternative survival models are preferred. We provide preliminary criteria that may be used by researchers reporting or reviewing similar studies. However, as mediation analysis is increasingly used in clinical research, a comprehensive set of reporting criteria must be more formally developed, with input from clinicians, healthcare researchers, journal editors and methodological experts. Such criteria will greatly benefit researchers seeking to report not only the “why” but also the “how” of their findings.

## Additional files

Additional file 1: Comprehensive search strategy. Description: this file contains the search terms and strategy using in our comprehensive search. (DOCX 21 kb)

Additional file 2: Included Studies. Description: this table contains the details of all published papers selected for study inclusion. (DOCX 42 kb)

## Abbreviations

Cox PH: Cox Proportional Hazards
SEM: Structural Equation Modelling
