# Supplementary material for: Mediation analysis with a time-to-event outcome: a review of use and reporting in healthcare research
Source: BMC Med Res Methodol. 2018 Oct 29;18:118. doi: 10.1186/s12874-018-0578-7 (PMC6206666; doi:10.1186/s12874-018-0578-7)
Supplement: Supplementary file 1 — Comprehensive search strategy. Description: this file contains the search terms and strategy using in our comprehensive search. (DOCX 21 kb) [file 12874_2018_578_MOESM1_ESM.docx]

**Additional File 1: Comprehensive search strategy**

**The databases searched were**:

- Medline (database inception 1946)
- Medline in process and epub (database inception 1946)
- Embase (database inception 1947)
- Web of Science for cited reference searching of 5 key papers (database inception 1900)

All searches executed December 9, 2016, except for EMBASE and Web of Science executed on December 12^th^, 2016

Database: Ovid MEDLINE(R) <1946 to November Week 5 2016>

Search Strategy:

--------------------------------------------------------------------------------

1 (mediat* adj2 measure*).mp. (1194)

2 (mediat* adj2 model*).mp. (3098)

3 (mediat* adj2 anal*).mp. (5149)

4 (mediat* adj2 variable*).mp. (1637)

5 (mediat* adj2 effect*).mp. (50199)

6 (intervening adj2 variable*).mp. (595)

7 (intermedia* adj2 variable*).mp. (622)

8 (sobel adj2 test*).mp. (186)

9 (partial adj2 mediat*).mp. (820)

10 (full adj2 mediat*).mp. (359)

11 indirect effect*.mp. (11146)

12 (moderat* adj2 mediat*).mp. (2139)

13 1 or 2 or 3 or 4 or 5 or 6 or 7 or 8 or 9 or 10 or 11 or 12 (72137)

14 exp survival analysis/ (251714)

15 (anal* adj2 surviv*).mp. (147262)

16 (cox adj2 model*).mp. (16013)

17 (failure adj2 time).mp. (2664)

18 kaplan-meier.mp. (81104)

19 product-limit.mp. (862)

20 (proportional adj2 hazard*).mp. (90459)

21 (additive adj2 hazard*).mp. (109)

22 (hazard* adj2 model*).mp. (83982)

23 (hazard* adj2 ratio*).mp. (75896)

24 (time adj1 event).mp. (587)

25 (time adj1 outcome).mp. (314)

26 (surviv* adj2 model*).mp. (5149)

27 (parametric adj2 survival).mp. (397)

28 14 or 15 or 16 or 17 or 18 or 19 or 20 or 21 or 22 or 23 or 24 or 25 or 26 or 27 (335659)

29 13 and 28 (732)

30 animals/ not (animals/ and humans/) (4636432)

31 29 not 30 (608)

Database: Epub Ahead of Print and In-Process & Other Non-Indexed Citations Ovid MEDLINE(R) <December 08, 2016>

Search Strategy:

--------------------------------------------------------------------------------

1 (mediat* adj2 measure*).mp. (119)

2 (mediat* adj2 model*).mp. (653)

3 (mediat* adj2 anal*).mp. (1088)

4 (mediat* adj2 variable*).mp. (211)

5 (mediat* adj2 effect*).mp. (4312)

6 (intervening adj2 variable*).mp. (62)

7 (intermedia* adj2 variable*).mp. (51)

8 (sobel adj2 test*).mp. (38)

9 (partial adj2 mediat*).mp. (146)

10 (full adj2 mediat*).mp. (56)

11 indirect effect*.mp. (1755)

12 (moderat* adj2 mediat*).mp. (498)

13 1 or 2 or 3 or 4 or 5 or 6 or 7 or 8 or 9 or 10 or 11 or 12 (7595)

14 (anal* adj2 surviv*).mp. (4928)

15 (cox adj2 model*).mp. (2463)

16 (failure adj2 time).mp. (442)

17 kaplan-meier.mp. (6907)

18 product-limit.mp. (62)

19 (proportional adj2 hazard*).mp. (6194)

20 (additive adj2 hazard*).mp. (39)

21 (hazard* adj2 model*).mp. (4624)

22 (hazard* adj2 ratio*).mp. (11996)

23 (time adj1 event).mp. (110)

24 (time adj1 outcome).mp. (66)

25 (surviv* adj2 model*).mp. (715)

26 (parametric adj2 survival).mp. (84)

27 or/14-26 (23983)

28 13 and 27 (70)

Database: Embase <1974 to 2016 December 09>

Search Strategy:

--------------------------------------------------------------------------------

1 (mediat* adj2 measure*).mp. (1476)

2 (mediat* adj2 model*).mp. (3721)

3 (mediat* adj2 anal*).mp. (6310)

4 (mediat* adj2 variable*).mp. (1854)

5 (mediat* adj2 effect*).mp. (57622)

6 (intervening adj2 variable*).mp. (721)

7 (intermedia* adj2 variable*).mp. (588)

8 (sobel adj2 test*).mp. (303)

9 (partial adj2 mediat*).mp. (962)

10 (full adj2 mediat*).mp. (387)

11 indirect effect*.mp. (13078)

12 (moderat* adj2 mediat*).mp. (2408)

13 1 or 2 or 3 or 4 or 5 or 6 or 7 or 8 or 9 or 10 or 11 or 12 (83198)

14 exp survival analysis/ (2290)

15 (anal* adj2 surviv*).mp. (56703)

16 (cox adj2 model*).mp. (28142)

17 (failure adj2 time).mp. (3771)

18 kaplan-meier.mp. (90961)

19 product-limit.mp. (1222)

20 (proportional adj2 hazard*).mp. (127247)

21 (additive adj2 hazard*).mp. (141)

22 (hazard* adj2 model*).mp. (120422)

23 (hazard* adj2 ratio*).mp. (109760)

24 (time adj1 event).mp. (732)

25 (time adj1 outcome).mp. (561)

26 (surviv* adj2 model*).mp. (7285)

27 kaplan meier method/ (60553)

28 proportional hazards model/ (105469)

29 exp survival/ (923787)

30 (parametric adj2 survival).mp. (660)

31 or/14-30 (1049509)

32 13 and 31 (3290)

33 (exp animals/ or exp animal experimentation/ or nonhuman/) not ((exp animals/ or exp animal experimentation/ or nonhuman/) and exp human/) (5885848)

34 32 not 33 (2511)

35 limit 34 to (book or book series or chapter or conference abstract or conference paper or conference proceeding or "conference review") (982)

36 34 not 35 (1529)

Database: Web of Science Core Collection
Search date: December 12, 2016

Search Strategy:

--------------------------------------------------------------------------------

Key papers for Cited Reference Searching:

- PMC 21552129

- PMC 3204669

- PMID 25643116

- PMID 20954780

- PMID: 22781427

[Direct and Indirect Effects in a Survival Context](http://apps.webofknowledge.com.myaccess.library.utoronto.ca/full_record.do?product=WOS&search_mode=GeneralSearch&qid=7&SID=X2x3k274YRT3FCTjRKd&page=1&doc=1)

By: Lange, Theis; Hansen, Jorgen V.

[EPIDEMIOLOGY](javascript:;)   Volume: 22  Issue: 4   Pages: 575-581   Published: JUL 2011

Times Cited: [63](http://apps.webofknowledge.com.myaccess.library.utoronto.ca/CitingArticles.do?product=WOS&SID=X2x3k274YRT3FCTjRKd&search_mode=CitingArticles&parentProduct=WOS&parentQid=7&parentDoc=1&REFID=416201932&excludeEventConfig=ExcludeIfFromNonInterProduct)

PMC 21552129
(from Web of Science Core Collection)

[On Causal Mediation Analysis with a Survival Outcome](https://www.ncbi.nlm.nih.gov/pmc/articles/PMC3204669/)

Eric J. Tchetgen Tchetgen

Int J Biostat. 2011 Jan 1; 7(1): 33. Published online 2011 Sep 2. doi: 10.2202/1557-4679.1351

Times Cited: [8](http://apps.webofknowledge.com.myaccess.library.utoronto.ca/CitingArticles.do?product=WOS&SID=X2x3k274YRT3FCTjRKd&search_mode=CitingArticles&parentProduct=WOS&parentQid=12&parentDoc=1&REFID=423931936&excludeEventConfig=ExcludeIfFromNonInterProduct)

PMC3204669
(from Web of Science Core Collection)

[SAS Macro for Causal Mediation Analysis with Survival Data](http://apps.webofknowledge.com.myaccess.library.utoronto.ca/full_record.do?product=WOS&search_mode=GeneralSearch&qid=16&SID=X2x3k274YRT3FCTjRKd&page=1&doc=1)

By: Valeri, Linda; VanderWeele, Tyler J.

[EPIDEMIOLOGY](javascript:;)   Volume: 26   Issue: 2   Pages: E23-E24   Published: MAR 2015

Times Cited: [3](http://apps.webofknowledge.com.myaccess.library.utoronto.ca/CitingArticles.do?product=WOS&SID=X2x3k274YRT3FCTjRKd&search_mode=CitingArticles&parentProduct=WOS&parentQid=16&parentDoc=1&REFID=482193386&excludeEventConfig=ExcludeIfFromNonInterProduct)

PMID 25643116
(from Web of Science Core Collection)

A General Approach to Causal Mediation Analysis

By: Imai, Kosuke; Keele, Luke; Tingley, Dustin

PSYCHOLOGICAL METHODS Volume: 15 Issue: 4 Pages: 309-334 Published: DEC 2010

Times Cited: 347

PMID: 20954780

(from Web of Science Core Collection)

A Simple Unified Approach for Estimating Natural Direct and Indirect Effects

By: Lange, Theis; Vansteelandt, Stijn; Bekaert, Maarten

AMERICAN JOURNAL OF EPIDEMIOLOGY Volume: 176 Issue: 3 Pages: 190-195 Published: AUG 1 2012

Times Cited: 41

PMID 22781427

(from Web of Science Core Collection)
