# Supplementary material for: Mediation analysis with a time-to-event outcome: a review of use and reporting in healthcare research
Source: BMC Med Res Methodol. 2018 Oct 29;18:118. doi: 10.1186/s12874-018-0578-7 (PMC6206666; doi:10.1186/s12874-018-0578-7)
Supplement: Supplementary file 2 — Included Studies. Description: this table contains the details of all published papers selected for study inclusion. (DOCX 42 kb) [file 12874_2018_578_MOESM2_ESM.docx]

**Additional File 2. Included Studies**

| **First Author** | **Year** | **Title** | **Journal** | **Volume** |  | **Page Numbers** |
| --- | --- | --- | --- | --- | --- | --- |
| Amato, M. B. | 2015 | Driving pressure and survival in the acute respiratory distress syndrome | New England Journal of Medicine | 372 | 8 | 747-755 |
| Andersen, I. | 2004 | Do factors in the psychosocial work environment mediate the effect of socioeconomic position on the risk of myocardial infarction? Study from the Copenhagen Centre for Prospective Population Studies | Occupational & Environmental Medicine | 61 | 11 | 886-892 |
| Andersen, I. | 2005 | Income as mediator of the effect of occupation on the risk of myocardial infarction: does the income measurement matter? | Journal of Epidemiology & Community Health | 59 | 12 | 1080-1085 |
| Ariansen, I. | 2015 | Do repeated risk factor measurements influence the impact of education on cardiovascular mortality? | Heart | 101 | 23 | 1889-1894 |
| Assari, S. | 2016 | Number of Chronic Medical Conditions Fully Mediates the Effects of Race on Mortality; 25-Year Follow-Up of a Nationally Representative Sample of Americans | Journal of Racial & Ethnic Health Disparities | 20 |  | 20 |
| Barbour, K. E. | 2015 | Hip osteoarthritis and the risk of all-cause and disease-specific mortality in older women: A population-based cohort study | Arthritis and Rheumatology | 67 | 7 | 1798-1805 |
| Behrens, I. | 2016 | Association Between Hypertensive Disorders of Pregnancy and Later Risk of Cardiomyopathy | Jama-Journal of the American Medical Association | 315 | 10 | 1026-1033 |
| Berkowitz, S. A. | 2016 | Risk of Developing Diabetes Among Refugees and Immigrants: A Longitudinal Analysis | Journal of Community Health | 41 | 6 | 1274-1281 |
| Beydoun, M. A. | 2016 | Racial disparities in adult all-cause and cause-specific mortality among us adults: mediating and moderating factors | BMC Public Health | 16 | 1 | 1113 |
| Bjerregaard, L. G. | 2016 | Childhood height, adult height, and the risk of prostate cancer | Cancer Causes & Control | 27 | 4 | 561-567 |
| Bramsen, I. | 2007 | Wartime stressors and mental health symptoms as predictors of late-life mortality in World War II survivors | Journal of Affective Disorders | 103 | 03-Jan | 121-129 |
| Brenes, G. A. | 2005 | The influence of anxiety on the progression of disability | Journal of the American Geriatrics Society | 53 |  | 34-39 |
| Brock, T. M. | 2015 | Length of stay and its impact upon functional outcomes following lower limb arthroplasty | Knee Surgery, Sports Traumatology, Arthroscopy | 30 |  | 30 |
| Burns, R. A. | 2015 | Examination of the association between mental health, morbidity, and mortality in late life: findings from longitudinal community surveys | International Psychogeriatrics | 27 | 5 | 739-746 |
| Carney, R. M. | 2005 | Low heart rate variability and the effect of depression on post-myocardial infarction mortality | Archives of Internal Medicine | 165 | 13 | 1486-1491 |
| Cen, C. W. | 2012 | Social isolation, vital exhaustion, and incident heart failure: Findings from the Atherosclerosis Risk in Communities Study | European Journal of Heart Failure | 14 | 7 | 748-753 |
| Chatterjee, R. | 2014 | Non-traditional risk factors are important contributors to the racial disparity in diabetes risk: The atherosclerosis risk in communities study | Journal of General Internal Medicine | 29 | 2 | 290-297 |
| Chatterjee, R. | 2013 | Effects of changes in potassium with valsartan use on diabetes risk: Nateglinide and Valsartan in Impaired Glucose Tolerance Outcomes Research (NAVIGATOR) trial | American Journal of Hypertension | 26 | 6 | 723-726 |
| Christ, S. L. | 2008 | Assessment of the effect of visual impairment on mortality through multiple health pathways: structural equation modeling | Investigative Ophthalmology & Visual Science | 49 | 8 | 3318-3323 |
| Christ, S. L. | 2014 | Longitudinal relationships among visual acuity, daily functional status, and mortality the salisbury eye evaluation study | JAMA Ophthalmology | 132 | 12 | 1400-1406 |
| Comber, H. | 2016 | Causes and outcomes of emergency presentation of rectal cancer | International Journal of Cancer | 139 | 5 | 1031-1039 |
| Contiero, P. | 2013 | Fasting blood glucose and long-term prognosis of non-metastatic breast cancer: a cohort study | Breast Cancer Research & Treatment | 138 | 3 | 951-959 |
| Corbett, K. | 2015 | Adult social position and sick leave: the mediating effect of physical workload | Scandinavian Journal of Work Environment & Health | 41 | 6 | 542-553 |
| Creswell, K. G. | 2015 | A test of the stress-buffering model of social support in smoking cessation: is the relationship between social support and time to relapse mediated by reduced withdrawal symptoms? | Nicotine & Tobacco Research | 17 | 5 | 566-571 |
| Dalton, A. M. | 2016 | Residential neighbourhood greenspace is associated with reduced risk of incident diabetes in older people: a prospective cohort study | BMC Public Health | 16 | 1 | 1171 |
| de Boer, M. R. | 2004 | Different aspects of visual impairment as risk factors for falls and fractures in older men and women | Journal of Bone & Mineral Research | 19 | 9 | 1539-1547 |
| DeGarmo, D. S. | 2009 | Evaluating mediators of the impact of the Linking the Interests of Families and Teachers (LIFT) multimodal preventive intervention on substance use initiation and growth across adolescence | Prevention Science | 10 | 3 | 208-220 |
| Deger, S. M. | 2014 | Obesity, diabetes and survival in maintenance hemodialysis patients | Renal Failure | 36 | 4 | 546-551 |
| Dhana, K. | 2016 | Metabolically Healthy Obesity and the Risk of Cardiovascular Disease in the Elderly Population | PLoS ONE [Electronic Resource] | 11 | 4 | e0154273 |
| Dismuke, C. E. | 2016 | Racial/Ethnic Disparities in VA Services Utilization as a Partial Pathway to Mortality Differentials Among Veterans Diagnosed With TBI | Global Journal of Health Science | 8 | 2 | 260-272 |
| Emdin, C. A. | 2016 | Blood Pressure and Risk of Vascular Dementia: Evidence From a Primary Care Registry and a Cohort Study of Transient Ischemic Attack and Stroke | Stroke | 47 | 6 | 1429-1435 |
| Erez, A. | 2015 | The association between cardiorespiratory fitness and cardiovascular risk may be modulated by known cardiovascular risk factors | American Heart Journal | 169 | 6 | 916-923.e911 |
| Esquirol, Y. | 2014 | Could occupational physical activity mitigate the link between moderate kidney dysfunction and coronary heart disease? | International Journal of Cardiology | 177 | 3 | 1036-1041 |
| Feinstein, L. | 2016 | Does cytomegalovirus infection contribute to socioeconomic disparities in all-cause mortality? | Mechanisms of Ageing and Development | 158 |  | 53-61 |
| Ferguson, S. G. | 2006 | Does reducing withdrawal severity mediate nicotine patch efficacy? A randomized clinical trial | Journal of Consulting & Clinical Psychology | 74 | 6 | 1153-1161 |
| Fox, M. P. | 2009 | Role of breastfeeding cessation in mediating the relationship between maternal HIV disease stage and increased child mortality among HIV-exposed uninfected children | International Journal of Epidemiology | 38 | 2 | 569-576 |
| Frederiksen, B. L. | 2009 | Do patient characteristics, disease, or treatment explain social inequality in survival from colorectal cancer? | Social Science & Medicine | 69 | 7 | 1107-1115 |
| Freeman, E. E. | 2005 | Visual acuity change and mortality in older adults | Investigative Ophthalmology & Visual Science | 46 | 11 | 4040-4045 |
| Fritz, J. | 2015 | Mediation analysis of the relationship between sex, cardiovascular risk factors and mortality from coronary heart disease: Findings from the population-based VHM&PP cohort | Atherosclerosis | 243 | 1 | 86-92 |
| Fritze, T. | 2016 | Hearing Impairment Affects Dementia Incidence. An Analysis Based on Longitudinal Health Claims Data in Germany | PLoS ONE [Electronic Resource] | 11 | 7 | e0156876 |
| Galfalvy, H. | 2009 | Lower CSF MHPG predicts short-term risk for suicide attempt | International Journal of Neuropsychopharmacology | 12 | 10 | 1327-1335 |
| Gangwisch, J. E. | 2013 | Sleep duration and risk for hypertension in women: results from the nurses' health study | American Journal of Hypertension | 26 | 7 | 903-911 |
| Garcia-Esquinas, E. | 2014 | Cadmium Exposure and Cancer Mortality in a Prospective Cohort: The Strong Heart Study | Environmental Health Perspectives | 122 | 4 | 363-370 |
| Gerrits, M. M. J. G. | 2014 | Pain, not chronic disease, is associated with the recurrence of depressive and anxiety disorders | BMC Psychiatry | 14 (1) (no pagination) | 187 |  |
| Giesinger, I. | 2014 | Association of socioeconomic position with smoking and mortality: the contribution of early life circumstances in the 1946 birth cohort | Journal of Epidemiology & Community Health | 68 | 3 | 275-279 |
| Gonzalez-Pinto, A. | 2008 | Impact of cannabis and other drugs on age at onset of psychosis | Journal of Clinical Psychiatry | 69 | 8 | 1210-1216 |
| Guo, Y. | 2015 | The relationships among individual and regional smoking, socioeconomic status, and oral and pharyngeal cancer survival: a mediation analysis | Cancer Medicine | 4 | 10 | 1612-1619 |
| Hadland, S. E. | 2012 | Young age predicts poor antiretroviral adherence and viral load suppression among injection drug users | AIDS Patient Care & Stds | 26 | 5 | 274-280 |
| Hagen, K. B. | 2006 | What mediates the inverse association between education and occupational disability from back pain?--A prospective cohort study from the Nord-Trondelag health study in Norway | Social Science & Medicine | 63 | 5 | 1267-1275 |
| Hall, K. S. | 2015 | Social discrimination, stress, and risk of unintended pregnancy among young women | Journal of Adolescent Health | 56 | 3 | 330-337 |
| Hanning, U. | 2016 | Structural brain changes and all-cause mortality in the elderly population-the mediating role of inflammation | Age | 20 |  | 20 |
| Harkko, J. | 2016 | Educational attainment, labour market position and mental ill health as pathways from adversities in adolescence to disability pension in early adulthood: A Finnish cohort study using register data | Scandinavian Journal of Public Health | 7 |  | 7 |
| Harkness, K. L. | 2014 | ACUTE AND CHRONIC STRESS EXPOSURE PREDICTS 1-YEAR RECURRENCE IN ADULT OUTPATIENTS WITH RESIDUAL DEPRESSION SYMPTOMS FOLLOWING RESPONSE TO TREATMENT | Depression and Anxiety | 31 | 1 | 1-8 |
| Hassan, M. | 2014 | Interplay of parathyroid hormone and aldosterone antagonist in prevention of heart failure hospitalizations in chronic kidney disease | Journal of the Renin-Angiotensin-Aldosterone System | 15 | 3 | 278-285 |
| Haukenes, I. | 2013 | Smokers' increased risk for disability pension: social confounding or health-mediated effects? Gender-specific analyses of the Hordaland Health Study cohort | Journal of Epidemiology & Community Health | 67 | 9 | 758-764 |
| Hayashi, T. | 2014 | Efficacy of HMG-CoA reductase inhibitors in the prevention of cerebrovascular attack in 1016 patients older than 75 years among 4014 type 2 diabetic individuals | International Journal of Cardiology | 177 | 3 | 860-866 |
| He, L. | 2016 | Genome-wide time-to-event analysis on smoking progression stages in a family-based study | Brain and Behavior | 6 | 5 |  |
| Hendriks, S. A. | 2016 | Pneumonia, Intake Problems, and Survival Among Nursing Home Residents With Variable Stages of Dementia in the Netherlands: Results From a Prospective Observational Study | Alzheimer Disease & Associated Disorders | 15 |  | 15 |
| Hiles, S. A. | 2015 | The role of inflammatory markers in explaining the association between depression and cardiovascular hospitalisations | Journal of Behavioral Medicine | 38 | 4 | 609-619 |
| Hill, P. L. | 2011 | Conscientiousness and longevity: an examination of possible mediators | Health Psychology | 30 | 5 | 536-541 |
| Holmegard, H. N. | 2016 | Sex Hormones and Ischemic Stroke: A Prospective Cohort Study and Meta-Analyses | Journal of Clinical Endocrinology & Metabolism | 101 | 1 | 69-78 |
| Hoogwegt, M. T. | 2013 | Exercise mediates the association between positive affect and 5-year mortality in patients with ischemic heart disease | Circulation. Cardiovascular Quality & Outcomes | 6 | 5 | 559-566 |
| Horvei, L. D. | 2016 | C-reactive protein, obesity, and the risk of arterial and venous thrombosis | Journal of Thrombosis & Haemostasis | 14 | 8 | 1561-1571 |
| Howard, G. | 2011 | Traditional risk factors as the underlying cause of racial disparities in stroke: Lessons from the half-full (empty?) glass. [] | Stroke A Journal of Cerebral Circulation. | 29 |  |  |
| Hua, M. | 2016 | Effect of ICU strain on timing of limitations in life-sustaining therapy and on death | Intensive Care Medicine | 42 | 6 | 987-994 |
| Huang, Y. T. | 2016 | Mediation effect of hepatitis B and C on mortality | European Journal of Epidemiology | 31 | 6 | 625-633 |
| Huang, Y. T. | 2015 | Integrative Analysis of Micro-RNA, Gene Expression, and Survival of Glioblastoma Multiforme | Genetic Epidemiology | 39 | 2 | 134-143 |
| Huang, Y. T. | 2016 | Mediation Analysis of Hepatitis B and C in Relation to Hepatocellular Carcinoma Risk | Epidemiology | 27 | 1 | 14-20 |
| Hughes, M. F. | 2016 | The Predictive Value of Depressive Symptoms for All-Cause Mortality: Findings From the PRIME Belfast Study Examining the Role of Inflammation and Cardiovascular Risk Markers | Psychosomatic Medicine | 78 | 4 | 401-411 |
| Hvidtfeldt, U. A. | 2012 | Quantifying Mediating Effects of Endogenous Estrogen and Insulin in the Relation between Obesity, Alcohol Consumption, and Breast Cancer | Cancer Epidemiology Biomarkers & Prevention | 21 | 7 | 1203-1212 |
| Hvidtfeldt, U. A. | 2013 | Educational Differences in Postmenopausal Breast Cancer - Quantifying Indirect Effects through Health Behaviors, Body Mass Index and Reproductive Patterns | Plos One | 8 | 10 |  |
| Jablonska, B. | 2012 | A national cohort study of parental socioeconomic status and non-fatal suicidal behaviour--the mediating role of school performance | BMC Public Health | 12 |  | 17 |
| Jacobs, S. | 2015 | Evaluation of various biomarkers as potential mediators of the association between DELTA5 desaturase, DELTA6 desaturase, and stearoyl-CoA desaturase activity and incident type 2 diabetes in the European Prospective Investigation into Cancer and Nutrition-Potsdam Study | American Journal of Clinical Nutrition | 102 | 1 | 155-164 |
| Jacobs, W. | 2014 | The Right Ventricle Explains Sex Differences in Survival in Idiopathic Pulmonary Arterial Hypertension | Chest | 145 | 6 | 1230-1236 |
| Jager, S. | 2015 | Association between the Fatty Liver Index and Risk of Type 2 Diabetes in the EPIC-Potsdam Study | Plos One | 10 | 4 |  |
| James, P. | 2016 | Exposure to greenness and mortality in a nationwide prospective cohort study of women | Environmental Health Perspectives | 124 |  | 1344-1352 |
| Janket, S. J. | 2014 | Number of teeth, C-reactive protein, fibrinogen and cardiovascular mortality: a 15-year follow-up study in a Finnish cohort | Journal of Clinical Periodontology | 41 | 2 | 131-140 |
| Judd, S. E. | 2013 | Dietary patterns are associated with incident stroke and contribute to excess risk of stroke in black Americans | Stroke | 44 | 12 | 3305-3311 |
| Kamstrup, P. R. | 2016 | Elevated Lipoprotein(a) Levels, LPA Risk Genotypes, and Increased Risk of Heart Failure in the General Population | JACC Heart Failure | 4 | 1 | 78-87 |
| Kanters, S. | 2013 | Increased mortality among HIV-positive men on antiretroviral therapy: survival differences between sexes explained by late initiation in Uganda | HIV/AIDS Research and Palliative Care | 5 |  | 111-119 |
| Karpa, M. J. | 2010 | Associations between hearing impairment and mortality risk in older persons: the Blue Mountains Hearing Study | Annals of Epidemiology | 20 | 6 | 452-459 |
| Karpa, M. J. | 2009 | Direct and indirect effects of visual impairment on mortality risk in older persons: The Blue Mountains Eye Study | Archives of Ophthalmology | 127 | 10 | 1347-1353 |
| Kershaw, K. N. | 2013 | Quantifying the contributions of behavioral and biological risk factors to socioeconomic disparities in coronary heart disease incidence: the MORGEN study | European Journal of Epidemiology | 28 | 10 | 807-814 |
| Kim, D. | 2010 | Neighborhood socioeconomic status and behavioral pathways to risks of colon and rectal cancer in women | Cancer | 116 | 17 | 4187-4196 |
| Kizer, J. R. | 2012 | Total and high-molecular-weight adiponectin and risk of incident diabetes in older people | Diabetes Care | 35 |  | 415-423 |
| Lam, B. L. | 2008 | Reported visual impairment and risk of suicide: the 1986-1996 national health interview surveys | Archives of Ophthalmology | 126 | 7 | 975-980 |
| Lampure, A. | 2016 | Associations between liking for fat, sweet or salt and obesity risk in French adults: a prospective cohort study | International Journal of Behavioral Nutrition & Physical Activity | 13 |  | 74 |
| Lee, K. S. | 2014 | Depressive symptoms, health-related quality of life, and cardiac event-free survival in patients with heart failure: a mediation analysis | Quality of Life Research | 23 | 6 | 1869-1876 |
| Lu, Y. | 2015 | Mediators of the Effect of Body Mass Index on Coronary Heart Disease Decomposing Direct and Indirect Effects | Epidemiology | 26 | 2 | 153-162 |
| Lu, Y. | 2016 | Genetic variants affecting telomere length are associated with the prognosis of esophageal squamous cell carcinoma in a Chinese population | Molecular Carcinogenesis | 6 |  | 6 |
| Lundin, H. | 2016 | High Serum Insulin-Like Growth Factor-Binding Protein 1 (IGFBP-1) is Associated with High Fracture Risk Independent of Insulin-Like Growth Factor 1 (IGF-I) | Calcified Tissue International | 99 | 4 | 333-339 |
| Manttari, M. | 1997 | Alcohol and coronary heart disease: the roles of HDL-cholesterol and smoking | Journal of Internal Medicine | 241 | 2 | 157-163 |
| McCandless, L. C. | 2015 | Criminal justice system contact and mortality among offenders with mental illness in British Columbia: an assessment of mediation | Journal of Epidemiology & Community Health | 69 | 5 | 460-466 |
| Minnis, A. M. | 2008 | Gang exposure and pregnancy incidence among female adolescents in San Francisco: evidence for the need to integrate reproductive health with violence prevention efforts | American Journal of Epidemiology | 167 | 9 | 1102-1109 |
| Morgan, R. O. | 2013 | Modeling causes of aggressive behavior in patients with dementia | Gerontologist | 53 | 5 | 738-747 |
| Morton, P. M. | 2016 | Childhood Misfortune, Personality, and Heart Attack: Does Personality Mediate Risk of Myocardial Infarction? | Journals of Gerontology Series B Psychological Sciences & Social Sciences | 12 |  | 12 |
| Muennig, P. | 2013 | Do the psychosocial risks associated with television viewing increase mortality? Evidence from the 2008 General Social Survey-National Death Index dataset | Annals of Epidemiology | 23 | 6 | 355-360 |
| Nordahl, H. | 2014 | Education and Cause-specific Mortality The Mediating Role of Differential Exposure and Vulnerability to Behavioral Risk Factors | Epidemiology | 25 | 3 | 389-396 |
| Nordahl, H. | 2013 | Education and risk of coronary heart disease: assessment of mediation by behavioral risk factors using the additive hazards model.[Erratum appears in Eur J Epidemiol. 2014 Apr;29(4):303-6] | European Journal of Epidemiology | 28 | 2 | 149-157 |
| Nummela, O. | 2012 | Trust, self-rated health and mortality: a longitudinal study among ageing people in Southern Finland | Social Science & Medicine | 74 | 10 | 1639-1643 |
| O'Connor, M. L. | 2013 | Mediators of the association between driving cessation and mortality among older adults | Journal of Aging & Health | 25 | 8 Suppl | 249S-269S |
| Oddo, V. M. | 2016 | Stunting Mediates the Association between Small-for-Gestational-Age and Postneonatal Mortality | Journal of Nutrition | 146 | 11 | 2383-2387 |
| Ojha, R. P. | 2014 | Younger age distribution of cervical cancer incidence among survivors of pediatric and young adult cancers | Gynecologic Oncology | 134 | 2 | 309-313 |
| Pan, W. C. | 2016 | Fine Particle Pollution, Alanine Transaminase, and Liver Cancer: A Taiwanese Prospective Cohort Study (REVEAL-HBV) | Journal of the National Cancer Institute | 108 | 3 |  |
| Park, A. N. | 2008 | Mediating factors in the relationship between income and mammography use in low-income insured women | Journal of Women's Health | 17 | 8 | 1371-1378 |
| Pence, B. W. | 2012 | Childhood trauma and health outcomes in HIV-infected patients: an exploration of causal pathways | Journal of Acquired Immune Deficiency Syndromes: JAIDS | 59 | 4 | 409-416 |
| Petrov, M. E. R. | 2014 | Self-reported Sleep Duration in Relation to Incident Stroke Symptoms: Nuances by Body Mass and Race from the REGARDS Study | Journal of Stroke & Cerebrovascular Diseases | 23 | 2 | E123-E132 |
| Qiao, Y. | 2015 | Impact of Alcohol Consumption on Substrate Remodeling and Ablation Outcome of Paroxysmal Atrial Fibrillation | Journal of the American Heart Association | 4 | 11 |  |
| Ras, R. T. | 2015 | Intake of phytosterols from natural sources and risk of cardiovascular disease in the European Prospective Investigation into Cancer and Nutrition-the Netherlands (EPIC-NL) population | European Journal of Preventive Cardiology | 22 | 8 | 1067-1075 |
| Rochon, J. | 2014 | Mediation analysis of the relationship between institutional research activity and patient survival | Bmc Medical Research Methodology | 14 |  |  |
| Royall, D. R. | 2008 | Clock-drawing potentially mediates the effect of depression on mortality: Replication in three cohorts | International Journal of Geriatric Psychiatry | 23 | 8 | 821-829 |
| Russell, E. | 2011 | Residential racial composition, spatial access to care, and breast cancer mortality among women in Georgia | Journal of Urban Health | 88 | 6 | 1117-1129 |
| Schairer, C. | 2016 | Quantifying the Role of Circulating Unconjugated Estradiol in Mediating the Body Mass Index-Breast Cancer Association | Cancer Epidemiology Biomarkers & Prevention | 25 | 1 | 105-113 |
| Schnurr, P. P. | 2000 | Physician-diagnosed medical disorders in relation to PTSD symptoms in older male military veterans | Health Psychology | 19 | 1 | 91-97 |
| Seven, E. | 2015 | Adipocytokines, C-reactive protein, and cardiovascular disease: a population-based prospective study | PLoS ONE [Electronic Resource] | 10 | 6 | e0128987 |
| Shafi, T. | 2008 | Changes in serum potassium mediate thiazide-induced diabetes.[Erratum appears in Hypertension. 2009 Feb;53(2):e19] | Hypertension | 52 | 6 | 1022-1029 |
| Shen, K. | 2014 | Direct and indirect effects of childhood conditions on survival and health among male and female elderly in China | Social Science and Medicine | 119 |  | 207-214 |
| Shiffman, S. | 2006 | Immediate hedonic response to smoking lapses: relationship to smoking relapse, and effects of nicotine replacement therapy | Psychopharmacology | 184 | 04-Mar | 608-618 |
| Showalter, T. N. | 2016 | Determinants of Quality Care and Mortality for Patients With Locally Advanced Cervical Cancer in Virginia | Medicine | 95 | 8 |  |
| Sieurin, J. | 2016 | Personality traits and the risk for Parkinson disease: a prospective study | European Journal of Epidemiology | 31 | 2 | 169-175 |
| Silhol, R. | 2011 | Investigating the spatial variability in incidence of coronary heart disease in the Gazel cohort: the impact of area socioeconomic position and mediating role of risk factors | Journal of Epidemiology & Community Health | 65 | 2 | 137-143 |
| Smith, P. M. | 2013 | Estimating the direct and indirect pathways between education and diabetes incidence among Canadian men and women: a mediation analysis | Annals of Epidemiology | 23 | 3 | 143-149 |
| Steel, J. L. | 2007 | Depression, immunity, and survival in patients with hepatobiliary carcinoma | Journal of Clinical Oncology | 25 | 17 | 2397-2405 |
| Stephens, A. S. | 2016 | Association of Gestational Age and Severe Neonatal Morbidity with Mortality in Early Childhood | Paediatric and Perinatal Epidemiology | 30 | 6 | 583-593 |
| Strijk, J. E. | 2011 | The role of work ability in the relationship between aerobic capacity and sick leave: a mediation analysis | Occupational & Environmental Medicine | 68 | 10 | 753-758 |
| Strong, D. R. | 2009 | Impact of bupropion and cognitive-behavioral treatment for depression on positive affect, negative affect, and urges to smoke during cessation treatment | Nicotine and Tobacco Research | 11 | 10 | 1142-1153 |
| Sudzinova, A. | 2016 | Does poorer self-rated health mediate the effect of Roma ethnicity on mortality in patients with coronary artery disease after coronaro-angiography? | International Journal of Public Health | 61 | 3 | 375-382 |
| Targownik, L. E. | 2013 | Inflammatory bowel disease and the risk of fracture after controlling for FRAX | Journal of Bone and Mineral Research | 28 |  | 1007-1013 |
| Turiano, N. A. | 2014 | Perceived Control Reduces Mortality Risk at Low, Not High, Education Levels | Health Psychology | 33 | 8 | 883-890 |
| Turiano, N. A. | 2012 | Smoking mediates the effect of conscientiousness on mortality: The Veterans Affairs Normative Aging Study | Journal of Research in Personality | 46 | 6 | 719-724 |
| van den Biggelaar, A. H. | 2004 | Long-term treatment of intestinal helminths increases mite skin-test reactivity in Gabonese schoolchildren | Journal of Infectious Diseases | 189 | 5 | 892-900 |
| Volk, M. L. | 2008 | Impact of the model for end-stage liver disease allocation policy on the use of high-risk organs for liver transplantation | Gastroenterology | 135 | 5 | 1568-1574 |
| Walter, R. B. | 2013 | Height as an Explanatory Factor for Sex Differences in Human Cancer | Jnci-Journal of the National Cancer Institute | 105 | 12 | 860-868 |
| Wang, A. L. | 2016 | Effects of leisure-time and occupational physical activities on 20-year incidence of acute myocardial infarction: mediation and interaction | Scandinavian Journal of Work Environment & Health | 42 |  | 423-434 |
| Wang, W. | 2016 | Forced Expiratory Volume in the First Second and Aldosterone as Mediators of Smoking Effect on Stroke in African Americans: The Jackson Heart Study | Journal of the American Heart Association | 5 | 1 |  |
| Warner, E. T. | 2015 | Racial and Ethnic Differences in Breast Cancer Survival: Mediating Effect of Tumor Characteristics and Sociodemographic and Treatment Factors | Journal of Clinical Oncology | 33 | 20 | 2254-2261 |
| Warner, V. | 2008 | The role of fear and anxiety in the familial risk for major depression: a three-generation study | Psychological Medicine | 38 | 11 | 1543-1556 |
| Wei, Y. | 2016 | A Missense Genetic Variant in LRRC16A/CARMIL1 Improves ARDS Survival by Attenuating Platelet Count Decline | American Journal of Respiratory & Critical Care Medicine | 21 |  | 21 |
| Weiss, A. | 2009 | Emotionally stable, intelligent men live longer: the Vietnam Experience Study cohort | Psychosomatic Medicine | 71 | 4 | 385-394 |
| Wijndaele, K. | 2011 | Television viewing and incident cardiovascular disease: prospective associations and mediation analysis in the EPIC Norfolk Study | PLoS ONE [Electronic Resource] | 6 | 5 | e20058 |
| Win, S. | 2011 | Depressive symptoms, physical inactivity and risk of cardiovascular mortality in older adults: The Cardiovascular Health Study | Heart | 97 | 6 | 500-505 |
| Wu, J. R. | 2012 | Medication adherence mediates the relationship between marital status and cardiac event-free survival in patients with heart failure | Heart & Lung | 41 | 2 | 107-114 |
| Wu, Y. T. | 2016 | Land use mix and five-year mortality in later life: Results from the Cognitive Function and Ageing Study | Health and Place | 38 |  | 54-60 |
| Yan, T. | 2013 | Exploring psychosocial pathways between neighbourhood characteristics and stroke in older adults: the cardiovascular health study | Age & Ageing | 42 | 3 | 391-397 |
| Yeboah, J. | 2014 | Mediation of Cardiovascular Risk Factor Effects Through Subclinical Vascular Disease The Multi-Ethnic Study of Atherosclerosis | Arteriosclerosis Thrombosis and Vascular Biology | 34 | 8 | 1778-+ |
| Yoshimasu, K. | 2016 | Mediating and Moderating Role of Depression, Conduct Disorder or Attention-Deficit/Hyperactivity Disorder in Developing Adolescent Substance Use Disorders: A Population-Based Study | PLoS ONE [Electronic Resource] | 11 | 6 | e0157488 |
| Zhang, Y. | 2013 | Organizational status of dialysis facilities and patient outcome: Does higher injectable medication use mediate increased mortality? | Health Services Research | 48 | 3 | 949-971 |
| Zheng, D. D. | 2012 | Increased mortality risk among the visually impaired: the roles of mental well-being and preventive care practices | Investigative Ophthalmology & Visual Science | 53 | 6 | 2685-2692 |
| Zheng, D. D. | 2014 | Visual acuity and increased mortality: the role of allostatic load and functional status | Investigative Ophthalmology & Visual Science | 55 | 8 | 5144-5150 |
